# Adenosine A_2A_ receptor inactivation alleviates early-onset cognitive dysfunction after traumatic brain injury involving an inhibition of tau hyperphosphorylation

**DOI:** 10.1038/tp.2017.98

**Published:** 2017-05-09

**Authors:** Z-A Zhao, Y Zhao, Y-L Ning, N Yang, Y Peng, P Li, X-Y Chen, D Liu, H Wang, X Chen, W Bai, J-F Chen, Y-G Zhou

**Affiliations:** 1 Molecular Biology Center, State Key Laboratory of Trauma, Burn, and Combined Injury, Research Institute of Surgery and Daping Hospital, Third Military Medical University, Chongqing, China; 2 Department of Neurosurgery, Research Institute of Surgery and Daping Hospital, Third Military Medical University, Chongqing, China; 3 Department of Neurology and Pharmacology, Boston University School of Medicine, Boston, MA, USA

## Abstract

Tau is a microtubule-associated protein, and the oligomeric and hyperphosphorylated forms of tau are increased significantly after neurotrauma and considered important factors in mediating cognitive dysfunction. Blockade of adenosine A_2A_ receptors, either by caffeine or gene knockout (KO), alleviates cognitive dysfunction after traumatic brain injury (TBI). We postulated that A_2A_R activation exacerbates cognitive impairment via promoting tau hyperphosphorylation. Using a mouse model of moderate controlled cortical impact, we showed that TBI induced hyperphosphorylated tau (p-tau) in the hippocampal dentate gyrus and spatial memory deficiency in the Morris water maze test at 7 days and 4 weeks after TBI. Importantly, pharmacological blockade (A_2A_R antagonist ZM241385 or non-selective adenosine receptor antagonist caffeine) or genetic inactivation of A_2A_Rs reduced the level of tau phosphorylation at Ser404 and alleviated spatial memory dysfunction. The A_2A_R control of p-tau is further supported by the observations that a KO of A_2A_R decreased the activity of the tau phosphorylation kinases, glycogen synthase kinase-3β (GSK-3β) and protein kinase A (PKA) after TBI, and by that CGS21680 (A_2A_R agonist) exacerbated okadaic acid-induced tau hyperphosphorylation in cultured primary hippocampal neurons. Lastly, CGS21680-induced neuronal tau hyperphosphorylation and axonal injury were effectively alleviated by individual treatments with ZM241385 (A_2A_R antagonist), H89 (PKA antagonist) and SB216763 (GSK-3β antagonist), or by the combined treatment with H89 and SB216763. Our findings suggest a novel mechanism whereby A_2A_R activation triggers cognitive dysfunction by increasing the phosphorylation level of tau protein after TBI and suggest a promising therapeutic and prophylactic strategy by targeting aberrant A_2A_R signaling via tau phosphorylation.

## Introduction

Traumatic brain injury (TBI) not only can lead to death but can also be a major cause of neurological and psychiatric disorders, such as headache,^1^ post-traumatic stress disorder,^2^ suicide^3^ and cognitive dysfunction^4^ in the chronic phase, even when the trauma is relatively mild.^5^ Blast exposure in combat, traffic accidents and high-contact sports such as boxing and American football are the main causes of TBI.^4, 6^ Nearly 20–30% of patients with Alzheimer’s disease (AD) or Parkinson's disease (PD) have a history of TBI, compared to only 8–10% of control subjects.^7^ Learning and memory impairments may occur several years or decades after TBI. Moreover, in many cases, this process is much faster and earlier than in other neurodegenerative diseases. AD-related lesions, such as hyperphosphorylated tau and amyloid-β accumulation, can be detected as early as 24 h after TBI in a mouse model.^8, 9^

Tau proteins belong to the microtubule-associated protein (MAP) family^10^ and can be subdivided into four regions: an N-terminal projection region, a proline-rich domain, a microtubule-binding domain (MBD) and a C-terminal region.^11^ The phosphorylation level of tau proteins is significantly increased in the brains of AD patients. Phosphorylated tau aggregates into paired helical filaments and forms neurofibrillary tangles (NFTs), the production of which is tightly correlated with the degree of cognitive impairment.^12, 13^ An increasing number of researchers believe that soluble hyperphosphorylated tau also directly damages microtubules.^14^ Tau protein has ~80 phosphorylation sites distributed in all four domains, and these sites are under the control of several kinases, such as PKA, GSK-3β and CDK-5. Several studies have found hyperphosphorylated tau in the brains of TBI patients,^5, 8, 15^ but why these pathological tau hyperphosphorylation events occur so rapidly and the reason for the early-onset cognitive dysfunction after TBI remain unclear.

Extracellular adenosine exerts physiological and pathological effects by acting at adenosine receptor (AR) subtypes (A_1_, A_2A_, A_2B_ and A_3_ receptors). Brain injury triggers a huge surge of extracellular adenosine and thus activates the A_2A_Rs.^16^ A_2A_Rs couple to members of the G protein family, and the canonical A_2A_R pathway involves the activation of cAMP/PKA.^17^ Our previous studies have shown that inactivation of A_2A_R genetically or pharmacologically by caffeine can ameliorate cognitive impairment and attenuate neuropathological damage induced by blast injury in mice;^4, 18^ chronic treatment with caffeine also has a similar protective effect accompanied by a reduction in cerebral edema and inflammatory cell infiltration.^19, 20^ It has also been reported that A_2A_R blockade prevents synaptotoxicity and memory dysfunction caused by β-amyloid peptides.^21^ A recent study indicated that A_2A_R deletion is protective in a mouse model of tauopathy.^22^ We therefore speculated that A_2A_Rs trigger TBI-induced cognitive impairment through activation of PKA, thereby inducing tau hyperphosphorylation and tau-related neuropathological damage.

In this study, we used an A_2A_R KO mouse model and caffeine, a non-selective antagonist of A_2A_R, to evaluate whether A_2A_R inactivation can attenuate the level of tau phosphorylation and alleviate cognitive impairment, as well as other types of neuropathological changes, at the chronic phase after TBI. We further explored the mechanism of tau phosphorylation by inhibiting the PKA-facilitated hyperphosphorylation of tau by GSK-3β.

## Materials and methods

### Animals

The A_2A_R knockout (KO) mice, established by gene targeting as previously described, and their wild-type littermates used in this study were from Dr Chen.^23, 24^ Congenic global A_2A_R KO mice on a C57BL/6J background were generated by backcrossing global A_2A_R KO mice on a mixed (129-Steel × C57BL/6J) genetic background with C57BL/6J mice for 13–15 generations. The mice were housed and maintained in a pathogen-free, temperature- and humidity-controlled room under a 12-h light/dark cycle at the Animal Care Center of the Research Institute of Surgery and Daping Hospital (Third Military Medical University, Chongqing, China). Male mice 2 to 3 months old (weighing 22 to 26 g) were used. Mice were allocated to SHAM and TBI groups according to random number table. All animal procedures were reviewed and approved by the Administration of Affairs Concerning Experimental Animals Guidelines of Third Military Medical University.

### Traumatic brain injury model

A moderate TBI model was produced by using the controlled cortical impact (CCI) method according to our previous protocol through measuring brain water content and neurological deficit scores.^19^ Briefly, mice were anesthetized with an intraperitoneal injection of 50 mg kg^−1^ pentobarbital sodium and then subjected to a 4–5-mm diameter craniotomy by using a motorized drill over the left parietal cortex, with the center between bregma and the lambdoid suture. We produced the CCI with an aerodynamic impact device (Brain Injury Device TBI-0310, PSI, Lexington, KY, USA) by using a metal tip with a 3-mm diameter. We set the parameters at 2 mm below the dura and an impact speed of 3.5 m s^−1^. An electric heating blanket was used to maintain the body temperature of mice until they were completely awake and were able to move freely ~3 h after the injury. Thickened bedding material was prepared to facilitate their food and water intake.

### Morris water maze test

The Morris water maze (MWM) test was carried out at 1 and 4 weeks after TBI as previously described.^4^ Briefly, the test includes three parts: a visible cue task, a reference memory task and a working memory task. Mice were subjected to a 2-day visible cue task (days −1 and −2) with a decorated platform and a training paradigm of 4 swimming trials per day for 4 consecutive days (days 1–4). The time spent searching for the hidden platform was recorded as the escape latency. Mice failing to find the platform within 60 s were guided by the experimenter to the platform, where they were allowed to stay for 20 s, and the escape latency was recorded as 60 s. A probe trial was performed on day 5. From days 6–8, the platform was moved to a new position every day (position of platform was fixed within the same day) to test working memory. We followed the four-trial ‘repeated acquisition protocol’ as previously described.^4, 25^ Briefly, mice were placed into the water at the same starting position opposite to the platform during all four trials and permitted to swim for up to 60 s until they located the platform. The time spent locating the platform was recorded as the escape latency, and the escape latency index ((trial 1−trial 4)/trial 1) was calculated to measure working memory.

### Neurologic deficit scoring and edema evaluation

Mice were scored for neurologic deficits before and 1, 3 and 6 days after TBI as described by Petullo *et al.*^26^ Briefly, neuromuscular function (forelimb flexion, torso twisting, lateral push, hindlimb placement, forelimb placement, inclined board, mobility), vestibulomotor function (balance beam) and complex neuromotor function (beam walk) were evaluated and scored with values of 0–1 or 0–2 in neuromuscular function, 0–6 in vestibulomotor function and 0–5 in complex neuromotor function. The total neurologic deficit score was equal to the summation of all scoring values. Cerebral water content was assayed using a wet–dry method at 24 h after TBI, as described previously.^27^ Briefly, ipsilateral injured cortices were immediately removed, weighed (wet weight) and placed in the thermostat at 80 °C for 48 h. Then, the cortices were weighed again (dry weight). The brain water content was determined as a percentage using the following equation: (wet weight–dry weight)/wet weight × 100. The schematic representation of this method and the process for the MWM, neurologic deficit scoring and edema evaluation are shown in Figure 3g.

### Primary culture of hippocampal neurons

Mouse hippocampal neuron cultures were prepared as previously described.^28^ In brief, embryos were obtained from 18-day gestating mice anaesthetized under pentobarbital sodium. The hippocampus was isolated using sterile microforceps under a stereomicroscope and treated with 0.25% trypsin for 15 min at 37 °C. Trypsinization was stopped by adding 10% FBS, and cell suspensions were seeded at 1 × 10^5^ cells cm^−^^2^ in Neurobasal medium (Invitrogen, Grand Island, NY, USA) containing 2% B27 supplement (Invitrogen), 0.5 mm l-glutamine and 25 μm l-glutamic acid. Half of the medium was replaced with B27/Neurobasal without l-glutamic acid 4 days later. The neurons were used after 14 days of culture.

### Immunofluorescence, immunohistochemistry and Golgi staining

After TBI, mice were perfused with saline and then with 4% paraformaldehyde. Brains were removed from the calvarium immediately and post-fixed in 4% paraformaldehyde. After fixation, coronal sections of 35 μm (cryosections) or 4 μm (paraffin sections) were cut and processed for immunohistochemistry and immunofluorescence. Sections were exposed to the indicated primary antibodies (Table 1). For immunofluorescence, the sections were then incubated with Cy3- or Alexa Fluor 488-conjugated secondary antibodies (Abcam, Cambridge, MA, USA; goat anti-mouse or goat anti-rabbit; 1:300). Nuclei were stained with 4′,6-diamidino-2-phenylindole (DAPI, Beyotime, Shanghai, China). The slices were then washed and mounted with UltraCruz mounting media (Santa Cruz Biotechnology, Dallas, TX, USA). For immunohistochemistry, after sequential incubation in 3% H_2_O_2_ and the primary antibody, sections were then incubated with biotin-conjugated secondary antibodies and visualized using a diaminobenzidine substrate kit (ZSGB-BIO, Beijing, China).The results were analyzed using Image-Pro Plus 4.5 (Media Cybernetics, Rockville, MD, USA) as described previously.^19, 29^ Measurements were performed on one field from each of three slices per mouse. For Golgi staining, the Rapid Golgi Stain Kit (FD NeuroTechnologies, Ellicot City, MD, USA) was used according to the manufacturer's instructions. In brief, the brains were immersed in impregnation solution for 2 weeks, and 80-μm sections were cut on a cryostat at −22 °C and stained for 10 min. Granule neurons in the contralateral dentate gyrus (DG) were selected. One or two dendritic segments from each neuron were chosen for quantification according to the criteria described by Chakraborti *et al.*^30^ For analysis of dendritic spine morphology, high-magnification images were captured using a camera (DFC290, Leica, Wetzlar, Germany) attached to an upright microscope (DM1000, Leica Microsystems). From each analyzed neuron, 3–5 dendritic segments, each at least 15 μm in length, were evaluated; 10 neurons were analyzed per brain.

### Western blot assays

To minimize the influence of anesthesia and hypothermia on tau phosphorylation,^31, 32^ mice were rapidly decapitated without anesthesia for western blot analysis. Western blot analysis was conducted using fresh and unfixed contralateral hippocampi obtained from mice at 7 days and 4 weeks after TBI. We also performed quantitative immunoblot analysis to measure the levels of phosphorylated tau in cultured primary hippocampal neurons. Hippocampal specimens were suspended in 0.4 ml of protease-phosphatase inhibitor buffer and homogenized in an ice-cold environment. The protease-phosphatase inhibitor buffer is a mixture of one pill of Protease and Phosphatase Inhibitor Mini Tablets (Pierce, Rockford, IL, USA) per 10 ml T-PERTM Tissue Protein Extraction Reagent (Pierce). Each tablet contains a mixture of several potent inhibitors including aprotinin, bestatin, E-64, leupeptin, sodium fluoride, sodium orthovanadate, sodium pyrophosphate, β-glycerophosphate and EDTA. After normalization, the samples were subjected to polyacrylamide gel electrophoresis (10% gel) and transferred onto an Immobilon-P PVDF membrane (Millipore, Billerica, MA, USA). Immunoblot analysis was performed to detect phosphorylation of tau at different sites, glial fibrillary acidic protein (GFAP, a marker of astrogliosis), and activation of PKA and GSK-3β. The primary antibodies used are listed in Table 1. After probing with horseradish peroxidase-conjugated secondary antibody, the membranes were visualized with SuperSignal Chemiluminescent Substrates (Pierce).

### Pharmacological treatments

We used OA to mimic the nocuous condition of TBI in the primary cultured hippocampal neurons. The neurons were exposed to OA (25 nm) for 12 h.To elucidate the signaling pathway associated with A_2A_R action, the A_2A_R agonist3-[4-[2-[[6-amino-9-[(2R,3R,4S,5S)-5-(ethylcarbamoyl)-3,4-dihydroxy-oxolan-2-yl]purin-2-yl]amino]ethyl]phenyl]propanoic acid (CGS21680) (100 nm)and/or the A_2A_R antagonist 4-(2-thyl)-phenol (ZM241385) (1 μm) was added 10 min before OA treatment.^33^ To explore the roles of PKA and GSK-3β in A_2A_R activation-induced tau hyperphosphorylation, the PKA inhibitorN-[2-[[3-(4-bromophenyl)-2-propenyl]amino]ethyl]-5-isoquinolinesulfonamide dihydrate dihydrochloride (H89, 10 μm) and/or the GSK-3β inhibitor 3-(2,4-dichlorophenyl)-4-(1-methyl-1H-indol-3-yl)-1H-pyrrole-2,5-dione (SB216763, 1 μm) was added 30 min before OA treatment. This experiment was repeated three times, and values are presented as the mean±s.e.m. Caffeine (a non-selective A_2A_R antagonist) was administered through the drinking water (0.25 g l^−1^) from 3 weeks before TBI and continuing until the mice were killed, as described previously.^19^

### Patients

We examined the brain tissues of three patients who were hospitalized for severe TBI with potentially fatal hematoma and required emergent surgical debridement and intracranial pressure control (refer to Table 2 for their detailed demographic characteristics). These brain tissue samples were trimmed into 5-mm thick blocks and plunged into 10% formaldehyde solution immediately after resectioning and were fixed for 24 h before further pathological examination. Written informed consent was obtained from all participants. All methods were performed in accordance with the protocols approved by the Third Military Medical University (No. ChiCTR-OPC-16008982) and the methods were carried out in accordance with the approved guidelines.

### Statistical analysis

Results are expressed as the mean±s.e.m. All semi-quantitative assessments of histological staining were made by a single investigator blinded to the genotype and treatment of the experimental animals. Sample size was chosen according to previous reports and our pre-experiments. Differences between two groups were analyzed using Student’s *t*-test or the rank sum test for discontinuous variables, and statistical comparisons of more than two groups were performed using factorial ANOVA followed by Bonferroni’s *post hoc* test. Two-way analyses of variance (ANOVAs) were used to assess the effects of genotype, day, and the genotype × day interaction in the MWM. The comparisons of neurologic deficit scores among groups were analyzed by Mann-Whitney *U*-test. A value of *P*<0.05 was considered statistically significant.

## Results

### Astrogliosis, edema and hyperphosphorylation of tau after TBI

**Figure 1:**
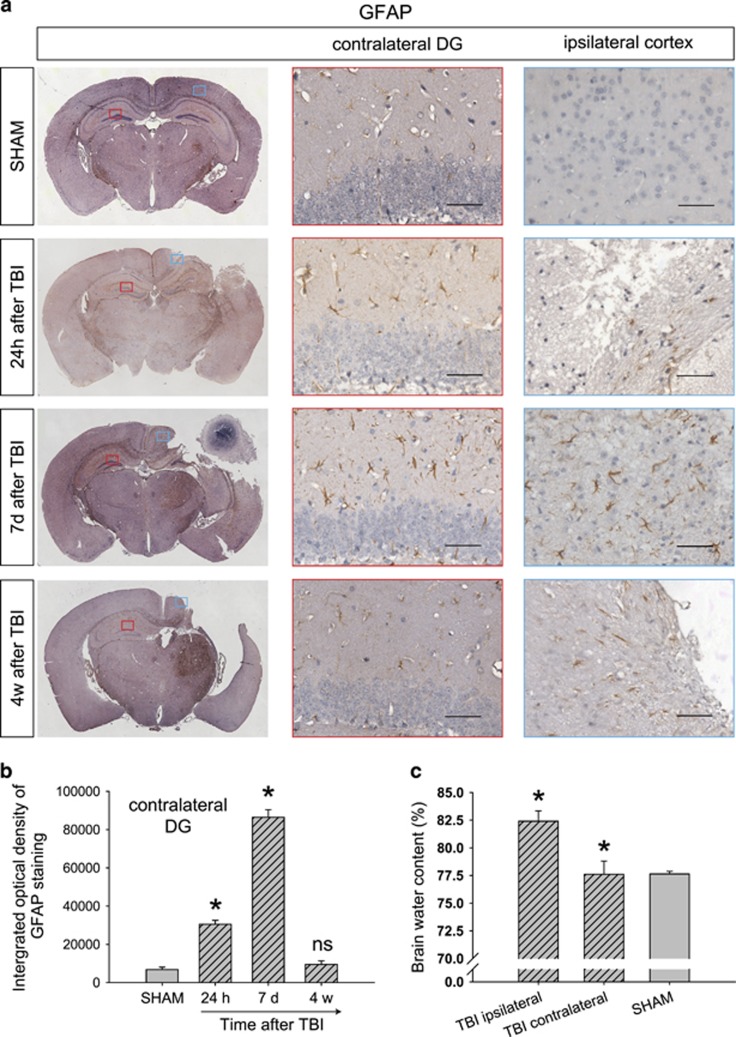
Astrogliosis and edema indicate the moderate degree of our traumatic brain injury (TBI) mouse model. (**a**) Astrogliosis was obvious in the contralateral dentate gyrus (DG) (red box) and the ipsilateral impacted cortex (blue box) at 24 h and 7 days after TBI. Scale bar, 50 μm. (**b**) Integrated optical density of positive GFAP staining in the contralateral DG. *n*=3 mice at each time point. Data represent mean±s.e.m., *P<0.01 compared to the SHAM group, ^ns^*P*>0.05 compared to the SHAM group, one-way ANOVA. (**c**) Brain water content after TBI; *n*=6 per group. Data represent mean±s.e.m., *P<0.01 compared to the SHAM group, one-way ANOVA.

In the contralateral DG and ipsilateral cortex around the impacted region, astrogliosis was obvious 24 h and 7 days after TBI (Figures 1a and b). We analyzed the GFAP immunoreactivity in the contralateral DG and found an increase in GFAP staining at 24 h after TBI. GFAP level continued to increase at 7 days and returned to relatively normal level at 4 weeks after TBI (Figure 1b). The area of lost tissue expanded gradually, and the hippocampus beneath the area of impact was badly damaged at 7 days after TBI and almost disappeared at 4 weeks after TBI (Figure 1a). The brain water content of the SHAM group was 77.6±0.1%. Consistent with our previous study,^20, 33^ the brain water content of the injured cortex increased to 82.4±0.4%, and that of the contralateral cortex was 77.6±0.4%, similar to the SHAM group (Figure 1c).

**Figure 2:**
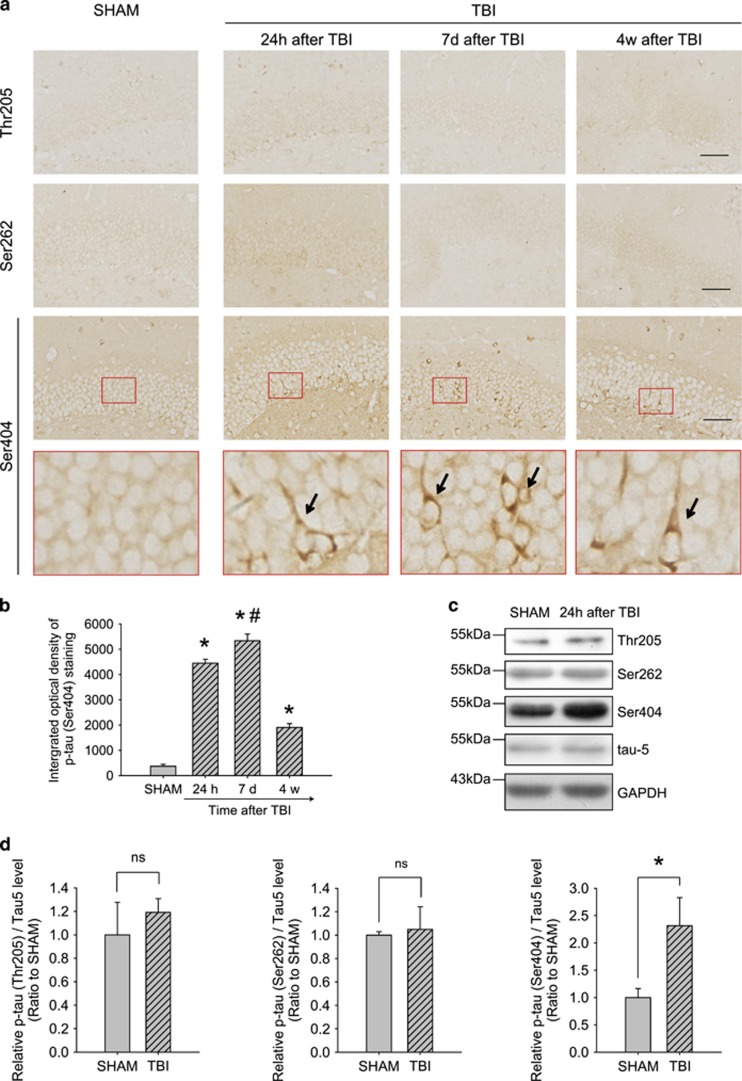
Hyperphosphorylation of tau in wild-type mice after traumatic brain injury (TBI). (**a**) The contralateral dentate gyrus (DG) showed a significant increase in the level of tau phosphorylated at Ser404 at 24 h, 7 days and 4 weeks post-injury. The levels of tau phosphorylation at Thr205 and Ser262 did not change obviously. Scale bar, 50 μm. (**b**) The integrated optical density of Ser404 phospho-tau staining in the contralateral DG of SHAM mice and mice 24 h, 7 days and 4 weeks after TBI. *n*=3 per group. Data represent mean±s.e.m., *P<0.05 compared to the SHAM group, ^#^*P*<0.05 compared to the 24 h group, one-way ANOVA. (**c**) Western blot analysis indicated that only phosphorylation at Ser404 increased at 24 h after TBI. (**d**) Relative levels of phospho-tau/total tau based on western blot results for the contralateral hippocampus at 24 h after TBI; *n*=3 per group. Data represent mean±s.e.m., *P<0.05 between the two groups, two-tailed *t*-test.

Antibodies detecting phosphorylation of tau at Thr205, Ser262 and Ser404 were used to recognize three domains (proline-rich domain, MBD and C terminal), and a significant increase in the level of phospho-tau at Ser404 was observed at 24 h, 7 days and 4 weeks after TBI. These increases in phosphorylated tau were mainly located around the injured cortex and DG of the contralateral hippocampus. The levels of phospho-tau at the Thr205 and Ser262 did not change significantly (Figure 2). Owing to the influence of anesthesia on tau phosphorylation, we paid particular attention to the p-tau level in the SHAM group. Despite the existence of p-tau in the SHAM group, the significant increase in the p-tau levels after TBI indicates anesthesia does not influence the interpretation of our results.

### Inactivation of A2AR attenuated TBI-induced memory impairment and tau hyperphosphorylation

**Figure 3:**
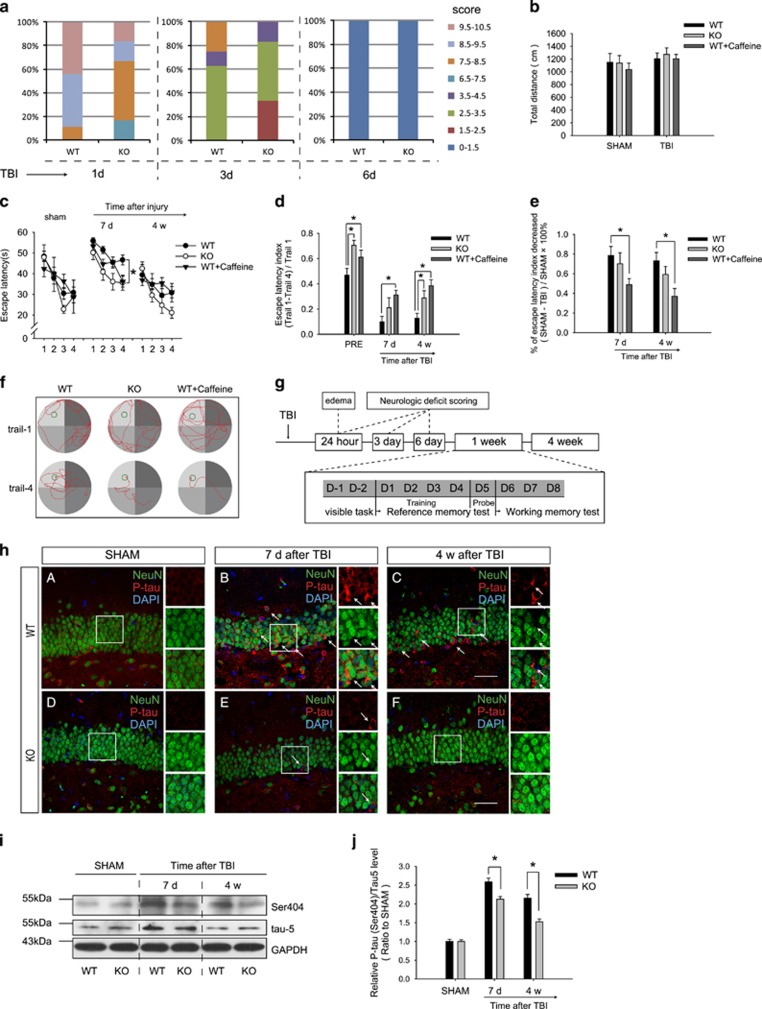
Inactivation of A_2A_ receptors attenuated spatial memory impairment and tau hyperphosphorylation after traumatic brain injury (TBI). (**a**) Neurological deficit scores at 1 day, 3 days and 6 days after TBI. The differences between genotypes were not significant at 24 h (*P*=0.662), 3 days (*P*=0.852) and 6 days (*P*=0.950) after TBI. *n*=6 per time point for knockout (KO) group, *n*=8 per time point for WT group, Mann–Whitney *U*-test. (**b**) All groups exhibited comparable total swimming distances on the first training day for the Morris water maze (MWM) (*P*>0.05, one-way ANOVA); *n*=25 in the WT group, *n*=12 in the KO group and *n*=12 in the WT+Caffeine group for each time point. Data represent mean±s.e.m. (**c**) Spatial reference memory was ameliorated in A_2A_R KO mice. There was a significant difference in the escape latency between the WT+TBI and KO TBI+TBI groups at 7 days after TBI (*P*<0.05). This tendency was also present but not significant at 4 weeks after TBI (*P*>0.05). Furthermore, there were no significant differences in the escape latency between the SHAM groups of WT and KO mice (*P*>0.05). Data represent mean±s.e.m., *P<0.05 between WT and KO groups, two-way ANOVA. (**d**) Spatial working memory impairment was also alleviated in A_2A_R KO mice compared with that of the WT group at 4 weeks after TBI. Data represent mean±s.e.m., *P<0.05, one-way ANOVA. (**e**) The recovery amplitude of spatial working memory. Data represent mean±s.e.m., *P<0.05, one-way ANOVA. (**f**) Representative swimming traces of mice at 4 weeks after TBI in the spatial working memory paradigm. (**g**) The schematic representation of the method and process for MWM, edema evaluation and neurologic deficit scoring. (**h**) Hyperphosphorylation of tau at Ser404 (red) was mainly observed in the granule neurons (green) in the contralateral DG. The level of tau phosphorylation at Ser404 increased significantly at 7 days (B) and 4 weeks (C) after TBI compared with that of the SHAM group (A). A_2A_R knockout attenuated this effect (E, F), scale bar, 50 μm. (**i**, **j**) A significant increase in tau phosphorylation at Ser404 in the contralateral hippocampus was also detected, and A_2A_R knockout could also alleviate the hyperphosphorylation of tau at 7 days and 4 weeks after TBI. *n*=3 per group, data represent mean±s.e.m., *P<0.05, one-way ANOVA.

Neurological deficit scores were divided into eight categories, which were represented by different colors. The percentage of mice in each category is shown in Figure 3a. Neurological examination showed that mice of both WT and A_2A_R KO mice showed slightly abnormal behavior at 24 h after TBI, with a median score of 8.25 in the WT group and 8.00 in the A_2A_R KO group. The neurological deficits of both genotypes recovered gradually and returned to normal levels at 6 days after TBI (Figure 3a) and no significant differences between WT and A_2A_R KO groups were observed at 24 h, 3 and 6 days after TBI. In addition, no significant difference in swimming distance was observed between the two groups on the first day of the training trials (Figure 3b). A_2A_R KO had little effect on basal reference memory. In the spatial reference paradigm, both WT and A_2A_R KO mice showed a longer escape latency in the training trials than their corresponding sham groups at 7 days after TBI. However, reference memory was relatively preserved in A_2A_R KO mice, manifesting as a shorter escape latency than in WT mice (Figure 3c). In the spatial working memory paradigm, the escape latency index ((T1−T4)/T1) of both TBI groups was relatively lower than their respective sham groups. The KO mice exhibited significantly higher escape latency indexes at baseline and 4 weeks after TBI than the WT mice (Figure 3d). Representative swimming traces of mice at 4 weeks after TBI in the spatial working memory paradigm are shown in Figure 3f. The phosphorylation level of tau markedly increased after TBI in the contralateral DG and was obviously decreased by A_2A_R KO (Figure 3h). This effect was further verified by western blot analysis, and changes in the main band of tau are shown in Figures 3i and j.

### Chronic caffeine pretreatment attenuated dendritic-spine degeneration and cognitive dysfunction after TBI

**Figure 4:**
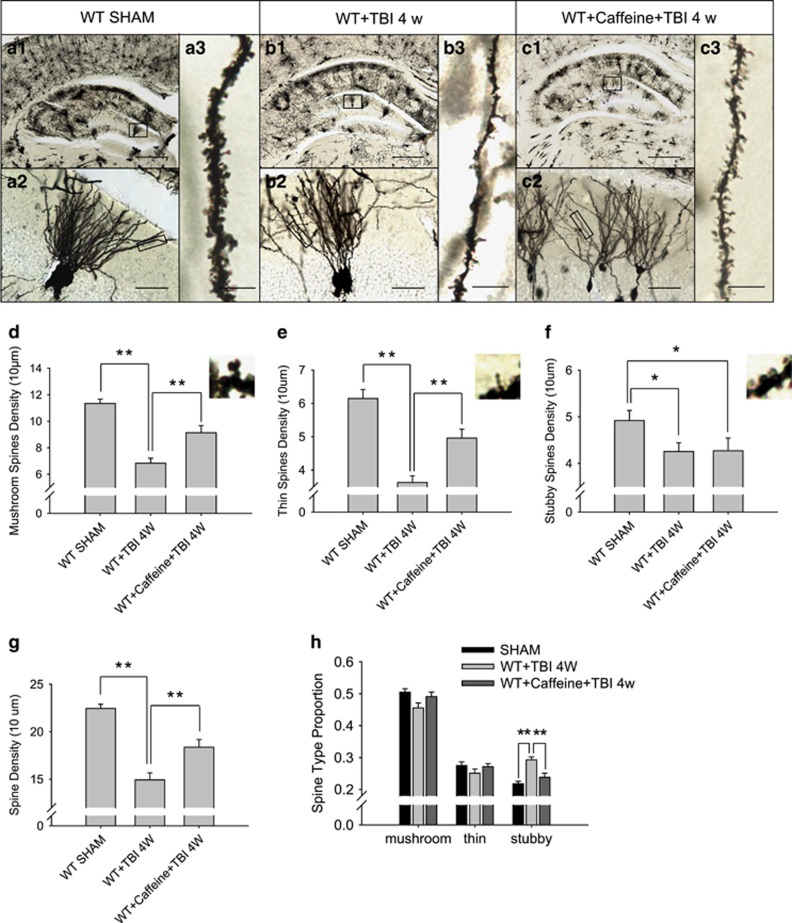
Chronic caffeine pretreatment attenuated the decrease in dendritic spine density in the dentate gyrus. Representative images of Golgi-impregnated dentate gyrus (DG) (**a1**, **b1**, **c1**), granule cells (**a2**, **b2**, **c2**) and dendritic spines (**a3**, **b3**, **c3**). Quantitative analysis of the densities of mushroom-shaped spines (**d**), thin spines (**e**) and stubby spines (**f**). (**g**) Quantitative analysis of the dendritic density of granule cells. (**h**) Changes in the proportions of spine morphological subtypes in the DG granule neurons. Data are presented as the mean±s.e.m. *n*=3 mice per group, *P<0.05, **P<0.01, one-way ANOVA. Scale bars represent 200 μm in **a1**–**c1**, 20 μm in **a2**–**c2** and 5 μm in **a3**–**c3**.

After 3 weeks of ingestion of caffeine, mice underwent TBI. Chronic caffeine ingestion rescued spatial working memory at 7 days and 4 weeks after TBI even more strongly than the knockout of A_2A_R (Figures 3d and e). Morphological analysis of dendritic spines showed that dendritic spine density was significantly reduced at 4 weeks after TBI and was obviously restored by chronic caffeine pretreatment (Figures 4a–h). Decreased densities of mushroom-shaped (Figure 4d), thin spines (Figure 4e) and total dendritic spine density (Figure 4g) were also significantly alleviated by caffeine pretreatment. In addition, although no significant difference in the density of stubby spines was observed (Figure 4f), caffeine prevented an increase in the proportion of stubby spines (Figure 4h).

### PKA activated by A2AR facilitated the phosphorylation of tau by GSK-3β

**Figure 5:**
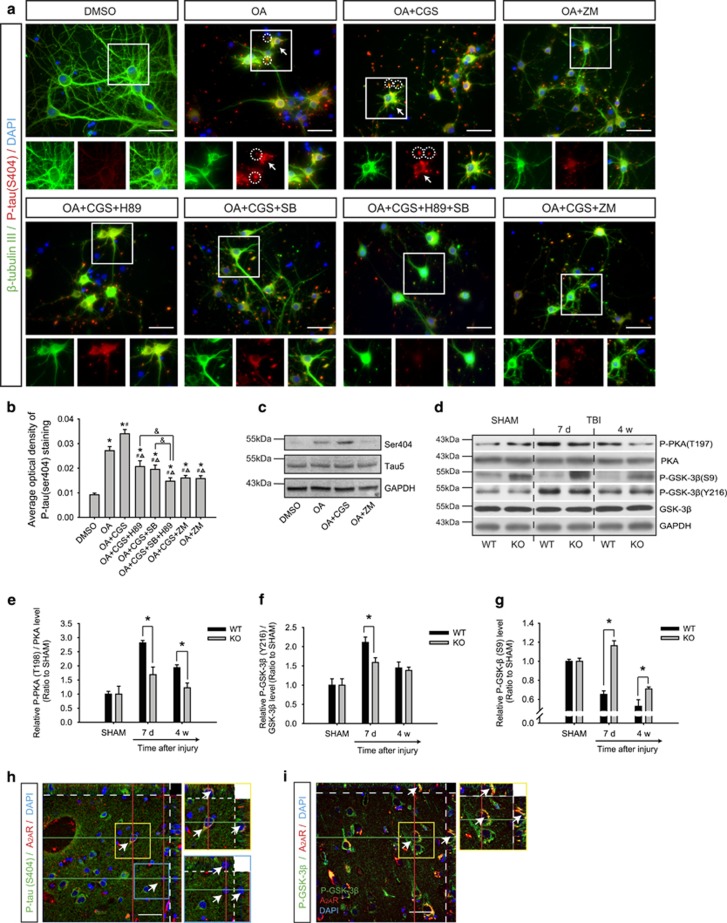
PKA and GSK-3β were involved in the neurotoxic effects of okadaic acid (OA) and CGS21680. (**a**) Treatment with OA for 12 h produced tau hyperphosphorylation at Ser404 (red) and significant decreases in axons and dendrites of primary hippocampal neurons (green). Increased p-tau signals were observed in the cell bodies (arrows) and the end of disconnected neurites (circles). CGS21680 treatment exacerbated OA-induced neuronal damage and tau hyperphosphorylation. H89 and/or SB216763 could alleviate the neurite loss and the hyperphosphorylation of tau at Ser404. ZM241385 by itself ameliorated OA-induced or OA+CGS-induced neuronal damage and tau hyperphosphorylation. Scale bar, 50 μm. CGS, CGS21680; SB, SB216763. (**b**) Levels of tau phosphorylation were measured using the average optical density of p-tau Ser404 staining. *P<0.05 compared with the DMSO group, ^#^*P*<0.05 compared with the OA group, ^△^*P*<0.05 compared with the OA+CGS group and ^&^*P*<0.05 between two groups, one-way ANOVA. (**c**) Western blot analysis indicating the level of tau phosphorylation after treatments with OA, CGS21680 and ZM241385. (**d**) Phosphorylation levels of PKA and GSK-3β were analyzed by western blot. (**e**) *In vivo* testing demonstrated significant increases in the p-PKA levels in the contralateral hippocampus at 7 days and 4 weeks after traumatic brain injury (TBI). However, PKA activity increased only slightly in A_2A_R KO mice. (**f**) Phosphorylation of GSK-3β at Y216 indicated that its activity did not change significantly at 4 weeks after TBI. (**g**) Inhibition of GSK-3β activity was observed in A_2A_R KO mice at 7 days and 4 weeks after TBI based on S9 phosphorylation. *n*=4 mice per group, data represent mean±s.e.m., *P<0.05, one-way ANOVA. (**h**) Co-localization of A_2A_R with p-tau (Ser404) (yellow box) in brain tissue samples from TBI patients. With no positive A_2A_R signal, no p-tau could be detected (blue box). (**i**) Co-localization of A_2A_R with p-GSK-3β (Y216) (yellow box). Scale bars, 50 μm.

Treatment of the primary cultured hippocampal neurons with OA (Figures 5a–c) and CGS21680 (Supplementary Figures S1a and b) separately for 12 h induced a significant increase in phosphorylated tau and a decrease in the number of neurites. These hyperphosphorylated tau proteins were mainly located in the end of disconnected neurites and the cell bodies. The p-tau level was further elevated by combined treatment with OA and CGS21680, and inhibition of PKA by H89 or inhibition of GSK-3β by SB216763 were able to alleviate the hyperphosphorylation of tau. The combination of H89 and SB216763 produced a stronger protective effect on ameliorating the increase in the p-tau level. ZM241385 alone had a protective effect similar to that of the combination of H89 and SB216763 (Figures 5a–c).

Phosphorylation of the catalytic subunit of PKA is reported to be closely associated with its activation.^34, 35^ Tyrosine phosphorylation of GSK-3β is necessary for its functional activity,^36, 37, 38^ whereas phosphorylation at Ser9 exerts an inhibitory effect.^39^ The p-PKA (T198) level increased significantly at 7 days and 4 weeks after TBI compared with that of the SHAM group (Figures 5d and e). P-GSK-3β (Y216) increased 7 days after TBI (Figures 5d and f). The levels of both phospho-kinases were markedly reduced in KO+TBI mice (Figures 5d–f). The A_2A_R KO group also showed an increase in the level of p-GSK-3β at Ser9 compared with that of the WT SHAM and WT+TBI groups (Figures 5d and g).

To examine whether the results observed in the mouse TBI model also occur in human brains, we collected brain tissues from three severe TBI patients. Accordingly, tau hyperphosphorylation was also prominent in frontal, temporal or occipital lobe tissue surrounding the hematoma from TBI patients, which was indicated by immunofluorescence analyses. A majority of neurons with hyperphosphorylated tau also showed positive signals of A_2A_Rs. Correspondingly, neurons with weak A_2A_R immunoreactivity showed a lower p-tau level (Figure 5h). Furthermore, A_2A_R colocalized with phospho-GSK-3β (pY216) in neurons of the injured cortex (Figure 5i).

## Discussion

### TBI triggers an early-onset memory impairment related to tau hyperphosphorylation

Phosphorylation of tau has site-specific effects,^40, 41^ and these sites work in concert to promote neurotoxicity *in vivo.*^42^ We checked several phosphorylation sites closely associated with neurodegeneration and found that Ser404 changed markedly, which maybe because Ser404 is one of the most favorable phosphorylation sites of GSK-3β. The hyperphosphorylaiton of tau at Ser404 of C-terminal promotes its self-aggregation markedly.^41^ P-tau that has dissociated from microtubules loses its microtubule assembly activity and can also aggregate with other normal MAPs on the microtubules.In this study, the phosphorylation level of tau at Ser404 increased significantly at 7 days and 4 weeks after TBI in the ipsilateral cortex and in the contralateral DG. The hippocampus is closely related to learning and memory, and the DG may have an important role in pattern separation (to differentiate between input cues).^43, 44^ Tissue loss of the ipsilateral cortex is unavoidable in our moderate TBI model; thus, the structural and functional maintenance of the contralateral hippocampus are necessary in the compensation process. Spatial reference memory and working memory, which are closely regulated by the hippocampus,^45^ were impaired at 7 days and 4 weeks after TBI. Neurological deficits and swimming distance on the first day of the training trials indicate that the cognitive impairment shown in the MWM did not result from motor dysfunction. Therefore, the temporal and spatial pattern of the hyperphosphorylated tau protein was consistent with the cognitive dysfunction observed in our TBI model. A previous study suggested that the post-traumatic tau pathology appeared to be independent of β-amyloid.^38^ On the basis of our results, we deduce that the hyperphosphorylation of tau is responsible for the rapid development of the spatial reference memory and working memory deficits in our TBI model.

### Inactivation of A2AR alleviated the TBI-induced cognitive dysfunction by decreasing the phosphorylation level of tau

Previous studies have revealed that inactivation of A_2A_Rs protects cognitive function under various conditions, including blast-TBI^4^ and amyloid-β-induced synaptotoxicity and tauopathy in an AD mouse model.^46^ A_2A_Rs are up-regulated upon various noxious brain conditions.^47, 48, 49, 50, 51^ In addition, we also detected an increase in A_2A_R expression in the mouse TBI model (data not shown) and TBI patients' brain tissues. Genetic or pharmacological inactivation of A_2A_R reduced tau hyperphosphorylation in the contralateral DG and alleviated memory impairment at 7 days and 4 weeks after TBI. In cultured primary hippocampal neurons, the level of OA-induced phosphorylation of tau at Ser404 was significantly increased after A_2A_R activation. Inactivation of A_2A_R reduced the OA- and CGS21680-induced hyperphosphorylation of tau. We demonstrate for we believe the first time that A_2A_R activation is closely related to TBI-induced impairments in spatial reference memory and working memory, and that the neurotoxic function of A_2A_R activation is probably mediated by the hyperphosphorylation of tau at Ser404.

### A_2A_R activation promotes hyperphosphorylation of tau at Ser404 through PKA and GSK-3β activation

Tau phosphorylation is controlled by more than 30 kinases. Different phosphorylation sites of tau have their own specific kinases, and some of the tau kinases regulate tau phosphorylation collectively at overlapping sites.^40, 42^ In our study, the level of Ser404 phosphorylation increased significantly at 7 days and 4 weeks after TBI. The level of phosphorylation at Ser404, which is often regarded as a GSK-3β site, increased significantly at 24 h, 7 days and 4 weeks after TBI. The cAMP-PKA pathway is the canonical pathway activated after A_2A_R activation, but its role in neurodegenerative diseases is still under debate.^51^ In recent years, some studies have demonstrated that over activation of PKA may induce cognitive dysfunction, and A_2A_R activation will ameliorate this effect.^52, 53, 54^ However, there is no evidence that PKA can directly phosphorylate Ser404 of tau protein, which is a target of GSK-3β.^55^ Interestingly, in our study, inactivation of either A_2A_R or PKA could decrease the level of phosphorylation at Ser404. Inactivation of GSK-3β decreased the level of Ser404 phosphorylation in primary hippocampal neurons previously treated with OA and CGS21680. However, the phosphorylation level of GSK-3β at Y216 did not change significantly. This phenomenon is consistent with previous reports that activation of PKA facilitated the phosphorylation of tau by GSK-3β at several sites, including Ser404.^56^ In addition, our results demonstrated that PKA was activated by A_2A_R after TBI.^33^ Thus, the regulatory effect of A_2A_R on tau may occur through the combined effects of PKA and GSK-3β. Notably, an increase in the level of p-GSK-3β (Ser9) was found in A_2A_R KO mice, indicating that A_2A_R deficiency is related to the inactivation of GSK-3β. It has been previously reported that inactivation of A_2A_R influenced the phosphorylation level of GSK-3β at Ser9 markedly in a mouse model of tauopathy.^22^ On the basis of the fact that the influences of GSK-3β on the hyperphosphorylation of tau have been well demonstrated, our present study provides strong evidence for the A_2A_R activation-induced tau phosphorylation and the resulting neurological dysfunction after TBI. It has been reported that Ser404 of tau is also under control of p38 (ref. 57) and CDK-5;^55^ whether A_2A_R could influence these two kinases and their influences on site-specific effects of tau phosphorylation after TBI need further investigation.

### Hyperphosphorylated tau is related to dendritic impairments after TBI

Dendritic degeneration^58^ are common features of TBI. In our previous study, neuron loss was observed in the chronic phase of TBI.^4^ In this study, we investigated the mechanism underlying the neurotoxic effect induced by hyperphosphorylated tau. Density and morphological analyses of dendritic spines revealed that granular neurons in the contralateral DG suffered degeneration despite their temporary survival. Dendritic spine protrusions have been conventionally classified as mushroom-shaped, thin or stubby. It has been reported that thin spines seem to be more plastic and are involved in learning, whereas mushroom spines have a larger role in memory.^59, 60, 61^ The morphological changes in spines are related to the strengths of their synaptic contacts and can reflect spine degeneration after TBI. Thus, the reduction in the fraction of mushroom-shaped spines and a marked increase in the proportion of stubby spines may contribute to the impairments in learning and memory following TBI.

### Caffeine consumption alleviates cognitive dysfunction and the morphological changes in dendritic spines after TBI

A series of longitudinal prospective studies have established an inverse relationship between caffeine consumption and the risk of developing cognitive impairments in aging and AD.^62, 63, 64^ Our previous studies and other studies have demonstrated the protective effect of caffeine on apoptosis, proinflammatory cytokine production and many other pathological changes in animal models.^19, 65^ It has been reported that A_2A_R blockade reverses hippocampal stress-induced electrophysiological impairment and dendritic atrophy.^66^ In our study, chronic caffeine consumption mimicked the protective effect of A_2A_R KO on spatial memory dysfunction, especially spatial working memory, after TBI. Meanwhile, caffeine attenuated the decreased density and morphological degeneration of dendritic spines. These results indicate that caffeine consumption is able to alleviate cognitive dysfunction after TBI.

## Conclusion

We demonstrate that A_2A_R activates PKA and facilitates the phosphorylation of tau by GSK-3β in the contralateral DG, and that this phosphorylation is crucial for the rapid development of cognitive dysfunction after TBI. These data provide experimental support for targeting A_2A_R as a preventative strategy against the early occurrence of cognitive dysfunction after TBI and possibly other neurodegenerative diseases. Meanwhile, we note that genetic knockout of A_2A_R or caffeine consumption mainly protected spatial working memory; future studies should focus on why hyperphosphorylated tau appears in specific brain regions and should address the mechanism underlying the selectivity of the protective effect of A_2A_R inhibition for certain types of memory.

## Figures and Tables

**Table 1:** Primary antibodies used in this study

*Name*	*Clonality*	*Origin*		*Vendor*	*Catalog number*	*Dilution*			
						*WB*	*IHC-P*		*IF/ICC*
*Tau*									
Tau antibody (TAU-5)	Mono		Mouse	Thermo Scientific	AHB0042	1:1000			
Phospho-tau pThr205 antibody	Poly		Rabbit	Thermo Scientific	OPA1-03153	1:1000	1:100		
Phospho-tau pSer262 antibody	Poly		Rabbit	Thermo Scientific	ab131354	1:1000	1:100		
Phospho-tau pSer404 antibody	Poly		Rabbit	Abcam	ab131338	1:1000	1:200		1:500
							
Anti-adenosine receptor A2a antibody	Mono		Mouse	Abcam	ab79714	1:1000	1:200		1:200
p-PKAα/β/γ cat (Thr198)	Poly		Rabbit	Santa Cruz Biotechnology	sc32968	1:500	1:100		
PKA	Poly		Goat	Santa Cruz Biotechnology	sc30668	1:500			
GSK-3β	Mono		Rabbit	Cell Signaling	9315	1:1000	1:100		
Anti-GSK-3β (phospho Y216)	Poly		Rabbit	Abcam	ab757475	1:1000	1:100		1:200
Anti-GSK-3β (phospho S9)	Mono		Rabbit	Abcam	ab75814	1:1000	1:100		
Anti-NeuN antibody	Mono		Rabbit	Abcam	ab177487		1:500		1:500
Anti-glial fibrillary acidic protein	Mono		Mouse	Millipore	MAB360		1:500		
Anti-GAPDH antibody	Poly		Rabbit	Abcam	ab37168	1:5000			
Anti-beta III tubulin antibody	Mono		Mouse	Abcam	ab78078				1:500

**Table 2:** Detailed demographic characteristics of TBI patients in our study

*Case no.*	*Age*	*Gender*	*Type of TBI*	*Symptoms before surgery*	*Presence of increased p-tau*
1	65	Female	Sever TBI on bilateral frontal cortex	Unconsciousness	Yes
2	58	Male	Sever TBI on right frontotemporal cortex	Unconsciousness	Yes
3	26	Male	Sever TBI on bilateral occipital cortex	Unconsciousness	Yes

Abbreviation: TBI, traumatic brain injury.
