# Supplementary figures and images for: Adenosine A2A receptor inactivation alleviates early-onset cognitive dysfunction after traumatic brain injury involving an inhibition of tau hyperphosphorylation
Source: Transl Psychiatry. 2017 May 9;7(5):e1123–. doi: 10.1038/tp.2017.98 (PMC5534966; doi:10.1038/tp.2017.98)

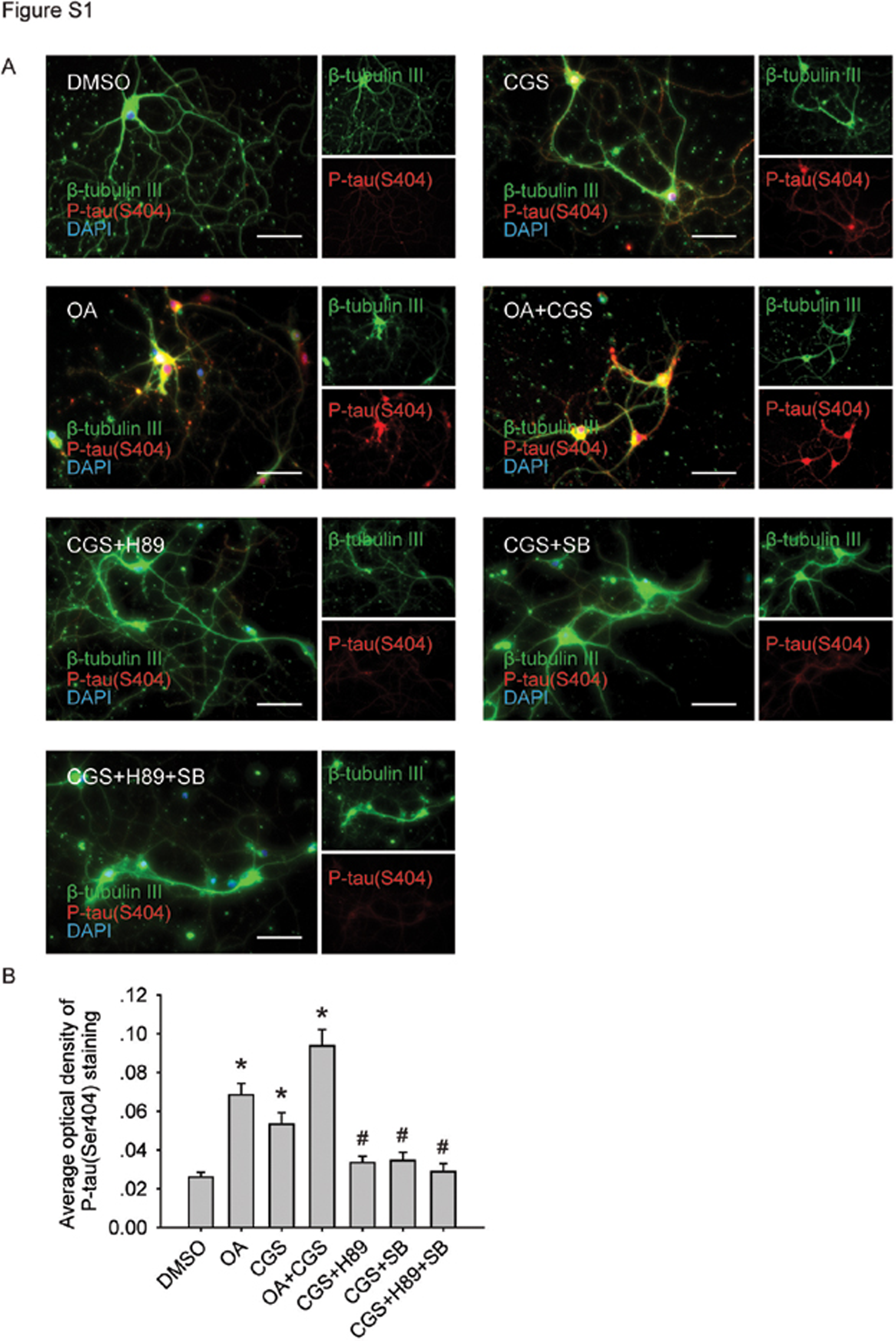

Supplement: Supplementary Figure 1 [file tp201798x1.tif]
