# Supplementary material for: Adenosine A2A receptor inactivation alleviates early-onset cognitive dysfunction after traumatic brain injury involving an inhibition of tau hyperphosphorylation
Source: Transl Psychiatry. 2017 May 9;7(5):e1123–. doi: 10.1038/tp.2017.98 (PMC5534966; doi:10.1038/tp.2017.98)
Supplement: Supplementary Figure 1 Legend [file tp201798x2.docx]

Figure S1. Activation of A_2A_Rs by CGS21680 increases p-tau level in vitro. (A) Treatment with OA and CGS21680 separately for 12 h produced a significant increase in the p-tau level at Ser404. The p-tau levels were further elevated by combined treatment with OA and CGS21680. Treatment with H89 and SB216763 separately attenuated the effect of CGS21680. Combined treatment with H89 and SB216763 further decreased the p-tau accumulation which was induced by CGS21680. Scale bar=50 μm. CGS, CGS21680; SB, SB216763. (B) Levels of tau phosphorylation were measured using the average optical density of p-tau Ser404 staining. Data represent mean ± s.e.m., *P<0.05 compared with the DMSO group, ^#^P<0.05 compared with the CGS group, one-way ANOVA.
